# Safety and Effects of a Four-Week Preoperative Low-Load Resistance Training With Blood Flow Restriction on Pre- and Postoperative Quadriceps Strength in Patients Undergoing Total Knee Arthroplasty: A Single-Blind Randomized Controlled Trial

**DOI:** 10.7759/cureus.64466

**Published:** 2024-07-13

**Authors:** Yusuke Kubo, Daisuke Fujita, Shuhei Sugiyama, Rie Takachu, Takeshi Sugiura, Masahiro Sawada, Kohtaro Yamashita, Kaori Kobori, Makoto Kobori

**Affiliations:** 1 Department of Rehabilitation Medicine, Kobori Orthopedic Clinic, Hamamatsu, JPN; 2 Department of Physical Therapy, Fukuoka International University of Health and Welfare, Fukuoka, JPN

**Keywords:** postoperative recovery, blood flow restriction, low-load resistance training, preoperative rehabilitation, quadriceps strength, total knee arthroplasty

## Abstract

Background/Objectives

Enhancing preoperative quadriceps strength and mitigating quadriceps strength loss due to total knee arthroplasty (TKA) is crucial for post-TKA recovery. This study compared the safety and effect of a four-week preoperative regimen of low-load resistance training with blood flow restriction (LLRT-BFR) with those of low-intensity resistance training with slow movement and tonic force generation (LST) on the pre- and postoperative quadriceps strength in patients undergoing TKA.

Methods

In this randomized controlled trial, 22 patients were assigned to either the LLRT-BFR (n=11) or LST (n=11) group. Primary outcomes included changes in quadriceps strength before and after the intervention and surgery. To assess safety, we monitored D-dimer and high-sensitivity C-reactive protein levels pre- and post-intervention. Statistical analysis involved independent samples *t*-tests and Mann-Whitney U tests for group comparisons of quadriceps strength changes. Additionally, a two-way repeated-measures analysis of variance was used to assess safety parameters.

Results

No significant differences were observed between the BFR and LST groups in terms of the rate of increase in quadriceps strength pre- and post-intervention (BFR: median 12.1%, interquartile range −0.8% to 19.5%; LST: median 6.2%, interquartile range 2.7% to 14.7%; p>0.99) or in the rate of reduction in quadriceps strength pre- and post-surgery (BFR: mean −72.4%, standard deviation ±11.2%; LST: mean −75.3%, standard deviation ±12.2%; p=0.57). Safety assessments showed no significant main effects of time, group, or interaction on the safety parameters (all p>0.05).

Conclusions

LLRT-BFR and LST demonstrated comparable effects on quadriceps strength before and after intervention and surgery in patients undergoing TKA. The lack of significant changes in the safety parameters supports the safety profile of both interventions, indicating their suitability for preoperative conditioning in patients scheduled for TKA.

## Introduction

Individuals who have undergone total knee arthroplasty (TKA) often experience marked quadriceps muscle weakness in the immediate postoperative period, which can continue for over a year [1,2]. Postoperative quadriceps weakness can negatively impact clinical outcomes by impairing walking ability, increasing fall risks, and reducing patient satisfaction [3-5]. Therefore, enhancing quadriceps strength recovery after surgery is essential for optimizing clinical outcomes in the postoperative phase.

Recently, a meta-analysis highlighted the efficacy of preoperative exercise interventions, known as prehabilitation, for accelerating and optimizing postoperative recovery, including enhancing quadriceps strength [6]. High-intensity resistance training of the quadriceps muscle enhances its strength preoperatively and sustains higher strength levels postoperatively [7,8]. Moreover, employing a specific form of low-intensity resistance exercise, involving slow movements and tonic force generation (LST), may prove effective in alleviating quadriceps weakness induced by TKA, particularly when coupled with tourniquet usage [9]. Therefore, increasing quadriceps strength before surgery and preventing TKA-induced quadriceps weakness can promote postoperative quadriceps strength recovery.

TKA-induced quadriceps weakness is partly associated with oxidative stress and acute inflammation, which typically result from ischemia-reperfusion (IR) injuries associated with the use of a tourniquet [10,11]. IR injury can be mitigated by implementing preconditioning methods, such as ischemic exposure and exercise. These approaches introduce hypoxia or exercise stimuli to the body, thereby reducing the severity of the IR injury [12,13]. LST, characterized by sustained muscle contraction during low-intensity resistance training, which exposes exercising muscles to hypoxia, can be considered a form of exercise that incorporates elements of ischemia and exercise preconditioning [14,15]. Hence, LST can reduce oxidative stress and acute inflammation caused by IR injury and, consequently, the associated quadriceps weakness due to the use of a tourniquet during TKA. Utilizing high-intensity resistance training or exercise modalities such as LST, which integrate aspects of ischemic and exercise preconditioning, can be regarded as highly effective in enhancing postoperative quadriceps strength through rehabilitation strategies.

We focused on low-load resistance training with blood flow restriction (LLRT-BFR), which is particularly suitable for older adults with knee pain who are scheduled for TKA. This approach involves applying external pressure to the lower limbs to restrict blood flow during exercise, thereby enabling low-intensity training under reduced oxygen conditions [16]. Comparable to high-intensity training, LLRT-BFR has been recognized for its effectiveness in enhancing muscle strength, while affording exercising muscles adequate exposure to hypoxia without requiring heavy loads or necessitating conscious maintenance of muscle contraction during resistance exercises [17,18]. Thus, LLRT-BFR can enhance preoperative quadriceps strength and can mitigate the reduction in quadriceps strength due to IR injuries caused by tourniquet use, making it an optimal choice for patients scheduled for TKA.

Therefore, the primary objective of the present study was to investigate the effectiveness of a four-week preoperative LLRT-BFR program focusing on the quadriceps muscles in patients scheduled for TKA. We compared patients treated with this intervention with an active control group performing LST. The secondary objective was to evaluate the safety of the LLRT-BFR program preoperatively. We hypothesized that performing the LLRT-BFR program over four weeks preoperatively would be safe, and that, compared with LST, would better strengthen the preoperative quadriceps muscles and reduce post-TKA quadriceps weakness by enhancing antioxidant capacity and reducing acute inflammation. We considered that this combined effect would preserve greater postoperative quadriceps strength, leading to improved functional capabilities and patient-reported outcomes. This study's findings were previously presented as a meeting abstract at the 11th Congress of the Japanese Society of Musculoskeletal Physical Therapy, held from October 13 to 15, 2023.

## Materials and methods

Study design and participants

This assessor-blinded, randomized, controlled trial was conducted from September 2019 to December 2021 at a Japanese orthopedic clinic. The inclusion criteria for the study were patients aged 60-79 years with advanced knee osteoarthritis (OA) who were scheduled for unilateral TKA and could attend the clinic two to three times weekly before surgery and one to two times weekly after surgery. Exclusion criteria comprised medical conditions, such as femoral condyle necrosis, cancer, rheumatoid arthritis, cardiovascular diseases, venous thrombosis, respiratory disorders (except asthma), uncontrolled diabetes (hemoglobin A1c levels ≥7%), renal disorders, and psychiatric conditions, such as dementia. Additionally, patients with a history of knee surgery, blood fibrinolysis or coagulation abnormalities, a body mass index higher than 30 kg/m², active smoking, and limb sensory or motor impairments were excluded. Functional limitations, including significant exercise restrictions due to motor dysfunction or lower limb pain, opting for epidural anesthesia, planning to transfer to another hospital post-surgery, and prior participation in this study for TKA of the opposite knee were also considered exclusion criteria.

The study received ethical approval from the Seirei Christopher University Ethics Committee (approval no. 19019), and informed consent was obtained from all participants in line with the revised 2008 Helsinki Declaration. The trial was registered in the University Hospital Medical Information Network Clinical Trials Registry (UMIN000037981) and adhered to the CONSORT guidelines.

**Figure 1:**
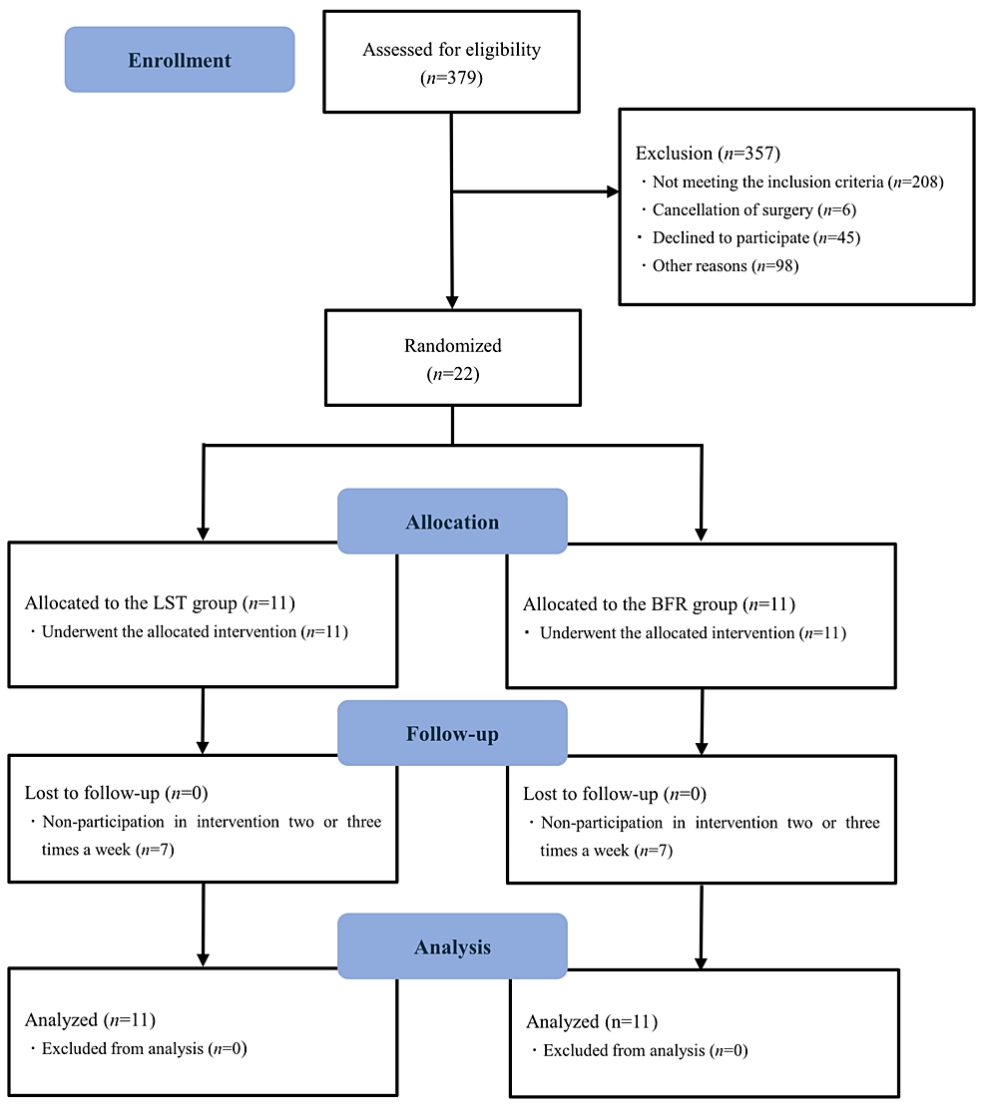
Flow diagram of progress through study phases LST, low-intensity resistance training with slow movements and tonic force generation; BFR, low-load resistance training with blood flow restriction.

Among 379 patients (Figure 1), 22 were included in the study, whereas 357 were excluded for the following reasons: 208 did not meet the inclusion criteria, six canceled their surgeries, 45 declined to participate due to concerns about the new coronavirus infection at the time, which led them to limit their outdoor activities, and 98 were excluded for other reasons. The active control (LST group) and intervention (BFR group) groups included 11 participants each. All included participants (two men and 20 women) successfully completed the study protocol.

Randomization

This study employed blocked randomization to ensure evenly distributed participant assignment between the BFR and LST groups, categorized by sex and age (in decades), with block sizes of two, four, or six. A physiotherapist who was blinded to the study’s data collection and analysis was responsible for generating the randomized sequence. We utilized an assessor-blinded approach to mitigate outcome evaluation bias. Two physical therapists were designated as assessors and were unaware of the participants’ group allocations. Furthermore, they were not involved in the supervision of training sessions.

Surgical procedures and perioperative care

All participants were admitted on the day of surgery and were discharged seven days later. They underwent tricompartmental uncemented TKA using a low-contact-stress implant (LCS Complete; DePuy, Johnson & Johnson Co., New Brunswick, NJ, USA) through a midvastus approach, which was performed by two skilled surgeons. Before closing the incision, 1000 mg of tranexamic acid was applied directly to the surgical area. A tourniquet (ATS 2000; Zimmer, Dover, OH, USA) was placed on the upper thigh and inflated to 300 mmHg. Anesthesia management during and after surgery, along with postoperative pain control, followed the protocols outlined in our previous study [19]. A wound drainage system was installed and removed 48 hours post-surgery. Blood transfusion was considered unnecessary.

Intervention

Both groups underwent interventions before and after surgery at a Japanese orthopedic clinic supervised by two experienced physiotherapists. Participants completed two to three training sessions per week for four weeks before surgery, although some could only complete one session per week. During the five days of hospitalization (excluding holidays and the day of discharge), two to three training sessions were conducted daily (totaling 60-90 min/day). After discharge, the participants trained one to two times per week for a period of three months.

Before surgery, both groups initially underwent thermotherapy involving a 10-minute hot pack treatment, followed by active and passive range-of-motion exercises to improve knee mobility, both administered by physiotherapists. Subsequently, they participated in three types of resistance and aerobic exercises using a cycle ergometer, maintaining a heart rate between 100 and 120 beats per minute for 10 minutes. The session concluded with a 10-minute electrotherapy treatment using an interferential current-delivering equipment. Each session lasted approximately 50 minutes. The resistance training components, including exercise types, intensity levels, sets, repetitions, and the duration of each repetition, were consistent across both groups. The BFR group underwent resistance training while using blood pressure cuffs (210 mm wide, 850 mm long, inflated to 100-120 mmHg) on their thighs and calves. In contrast, the LST group engaged in resistance training emphasizing sustained muscle contractions in each set. They used the same cuffs as those in the BFR group, but these were inflated to a lower pressure (20 mmHg), which was not high enough to restrict blood flow significantly; however, it simulated the BFR group’s experience. Resistance exercises included squats, forward lunges, and seated bilateral knee extensions. Squats and lunges utilized body weight for resistance. Bilateral knee extensions were performed at 30% of the maximum isometric voluntary contraction using a specific bilateral knee extension machine (WT-L02, Minato Medical Science Co., Ltd., Osaka, Japan). The maximum contraction was measured at a 90° knee angle (0° was a full extension) using the same machine. The exercise comprised three sets of 10 repetitions, each including 3-s eccentric, 3-s isometric, and 3-s concentric phases. A rest period of 30 s was observed between sets, and a 60-s rest was allocated for transitioning between different resistance exercises.

Inpatient rehabilitation commenced on the morning of postoperative day 1. The regimen included physical therapy, featuring active and passive range-of-motion exercises, quadriceps setting exercises combined with neuromuscular electrical stimulation, and exercises aimed at enhancing daily living activities, such as transitioning from sitting to standing and stair climbing. Walking exercises also commenced on the first day postoperatively, initially utilizing a four-wheeled walker and transitioning to T-handle canes by postoperative day 4 or 5. Cold therapy, when required, was administered for approximately 10 minutes multiple times daily.

From one week post-discharge to one month after surgery, both patient groups participated in an extensive rehabilitation program. This regimen included active and passive range-of-motion treatments, quadriceps-strengthening exercises enhanced by neuromuscular electrical stimulation, and gait training. Additionally, 10-minute sessions using hot packs and interferential current equipment were provided. During the second and third postoperative months, the LST and aerobic exercises performed preoperatively were reintroduced. The exercises were tailored to each patient’s physical state and pain tolerance. Furthermore, we offered guidance on home exercises, which included hip abductions, calf raises, bridge exercises, and stair climbing, and advised patients to complete three sets of 10 repetitions of each exercise daily.

Outcome measures

Participants completed seven assessment sessions. The initial baseline evaluation was conducted six weeks before surgery (6 W-Pre-Op), followed by a four-week intervention period. Subsequent assessments occurred one week before surgery (1 W-Pre-Op), immediately before the operation (Pre-Op), 24-hour post-surgery (24 h-Post-Op), four days post-surgery (4 D-Post-Op), one-month post-surgery (1 M-Post-Op), and at the final session, three months post-surgery (3 M-Post-Op). We evaluated morphological features, physical function, activities of daily living (ADL) performance, and quality of life (QOL) at 6 W-Pre-Op, 1 W-Pre-Op, 4 D-Post-Op, 1 M-Post-Op, and 3 M-Post-Op. The assessments included knee and thigh circumference (1 and 10 cm from the upper edge of the patella, respectively), isometric quadriceps strength, and knee pain during quadriceps strength testing. The functional tests comprised the 30-s chair stand test (30-s-CST), timed up-and-go (TUG) test, and 12-step stair-climbing test (SCT), with the SCT omitted only during the 4 D-Post-Op session. Additionally, we evaluated ADL scores using the Knee Injury and Osteoarthritis Outcome Score (KOOS-ADL) and Japanese Knee Osteoarthritis Measure (JKOM) scores, excluding the 4 D-Post-Op session.

To evaluate the effects of preoperative LLRT-BFR on oxidative stress levels and antioxidant capacity, diacron-reactive oxygen metabolites (d-ROMs) and biological antioxidant potential (BAP) were measured at 6 W-Pre-Op and Pre-Op. Additionally, to assess the effect of preoperative LLRT-BFR on acute postoperative inflammation, interleukin (IL)-6 levels were measured at Pre-Op and 24 h-Post-Op, as IL-6 levels can be influenced by IR injury [10]. This evaluation was based on studies that have reported the preventive effects of enhancing antioxidant capacity through ischemia and exercise preconditioning on acute inflammation caused by IR injury [13,20]. Moreover, to assess the safety of the four-week LLRT-BFR intervention before surgery, we monitored coagulation responses (D-dimer) and inflammation (high-sensitivity C-reactive protein [hs-CRP]) at 6 W-Pre-Op and Pre-Op.

The rate of increase in quadriceps strength before and after the intervention was defined as the relative change in quadriceps strength from the value at 6 W-Pre-Op to the value at 1 W-Pre-Op. The rate of reduction in quadriceps strength before and after surgery was defined as the relative change in quadriceps strength from the value at 1 W-Pre-Op to the value at 4 D-Post-Op. Quadriceps muscle weakness immediately after TKA has been associated with the rate of increase in knee and thigh circumference and the rate of increase in knee pain before and after surgery [9]. The relative rates of change in knee and thigh circumference from 1 W-Pre-Op to 4 D-Post-Op were calculated and defined as knee and thigh swelling measures, respectively. The relative rate of change was calculated using the formula:

Relative rate of change = (subsequent value - baseline value) / baseline value × 100.

The absolute change in knee pain from 1 W-Pre-Op to 4 D-Post-Op was calculated and defined as Δpain. The absolute change was calculated using the following formula:

absolute change = (subsequent value - baseline value).

The primary outcome focused on the rate of change in quadriceps strength before and after the intervention and surgery. Secondary outcomes included changes in d-ROM and BAP levels from before to after the intervention, as well as IL-6 levels from before to after surgery.

Knee and thigh circumferences

Knee and thigh circumferences were measured with the participants lying in the supine position, ensuring that their knees were relaxed and fully extended. A half-cylinder support was placed under the heel to enhance comfort and facilitate easier measurement. During the assessment, the examiner stood adjacent to the leg and identified two specific measurement points situated on a line extending from the center of the base of the patella to the anterior superior iliac spine. The points were identified as 1 and 10 cm above the base of the patella, serving as references for the circumference measurement of the operated knee. A non-stretchable tape measure was employed for precise measurement and was tightly wrapped around each of these points. To ensure accuracy and consistency, each measurement was performed twice at each point, with the values recorded to the nearest 0.1 cm. Circumference measurements using a tape measure are reported to have excellent intra-rater and good inter-rater reliabilities [21,22].

Isometric quadriceps strength

Quadriceps strength was quantified using a pull-type hand-held dynamometer (Mobie; Sakai Medical Co., Ltd., Tokyo, Japan), following the methodology outlined in a previous study [9]. The strength assessment involved participants seated with their hips and knees positioned at 90° and 75°, respectively. To ensure stability, participants held the sides of the couch on which they sat. The protocol commenced with two initial practice trials, followed by three trials of maximum effort, interspersed by 30-s rest periods. The two highest values of the three valid trials were recorded, and the average of these two values was used in the subsequent analysis. Quadriceps strength was then calculated as the maximum voluntary torque normalized to body mass (Nm/kg), with consideration of each participant’s lower leg length as the external lever arm as well as their body weight. Similar quadriceps strength measurements have been reported to have excellent intra-rater and good inter-rater reliabilities [23].

Knee pain

To assess knee pain experienced during quadriceps testing, the participants rated their pain using a numeric rating scale immediately after the tests. The scale ranged from 0 (indicating no pain) to 10 (representing the worst imaginable pain). This scale facilitated the quantification of knee pain levels. Notably, the numeric rating scale has demonstrated excellent test-retest reliability for measuring knee pain levels in individuals with OA [24].

Performance test

The 30-s-CST involved a chair with a backrest, no armrests, and a seat height of 44 cm. The participants were seated with their feet at shoulder width, knees bent at approximately 90°, and arms crossed over their chests. The task involved standing completely, straightening the hips and knees, and sitting down fully on the chair. The goal was to perform as many full stand-and-sit cycles as possible within 30 seconds. The total number of repetitions was recorded. Participants received a score of 0 if they could not stand even once, including when trying to use their hands. This test was conducted twice, and the average values were used for analysis. This test has previously shown excellent test-retest reliability in patients with knee OA scheduled for primary TKA [25].

For the TUG test, the participants were seated on a 44-cm highchair. The test timed the participants from standing up, walking 3 m, turning around, and returning to a seated position, all performed while moving as fast as they felt safe to do. The use of arms for assistance in standing up was not permitted, although a T-handle cane was allowed if needed. The time from standing up to sitting down was precisely measured to the nearest hundredth of a second. This test was conducted twice, and the average values were used for analysis. This test has shown excellent test-retest reliability in patients with TKA [26].

In the SCT, the participants were timed while ascending and descending a 12-step staircase, with each step being 18 cm high and 27.5 cm deep. Starting at the base, the participants ascended the staircase, turned around at the top step, and descended the staircase, at a rate as quickly as they felt safe to do. A T-handle cane was allowed if necessary. The duration of the task was measured to the nearest hundredth of a second and was recorded upon returning to the start position. This test was conducted twice, and the average values were used for analysis. This test has previously shown excellent test-retest reliability in patients with knee OA scheduled for primary TKA [25].

KOOS-ADL

The KOOS is a patient-reported questionnaire consisting of 42 items across five subscales: pain (nine items), other symptoms (seven items), function in daily living (17 items), sports and recreation activities (five items), and knee-related QOL (four items). Each item within these subscales was evaluated on a 5-point Likert scale, ranging from 0 (indicating no problems) to 4 (indicating extreme problems). The overall scores were converted into a 0-100 scale, with higher scores signifying better knee function and lower scores reflecting more severe knee issues. In this study, we focused on the “function in daily living” (i.e., ADL) subscale of the KOOS to evaluate the effect of the condition of the knee on patients’ daily activities. The KOOS is reported to be a valid, reliable, and responsive outcome measure for total joint replacement [27].

JKOM scores

The JKOM is a self-assessment tool tailored for Japanese individuals with knee conditions comprising 25 items categorized into four domains: eight items address pain and stiffness in the knees, 10 items focus on conditions in daily life, five items pertain to general activities, and two items relate to health conditions. Each item is scored from 0 to 4 points. The total JKOM score ranges from 0 to 100, with lower scores representing a better knee-related QOL. The JKOM has adequate reliability and validity for evaluating clinical outcomes in Japanese patients with knee OA [28].

Blood evaluation

We outsourced the blood sample analysis for serum hs-CRP, D-dimer, and IL-6 levels to Medic (Shizuoka, Japan). For d-ROMs and BAP testing, the blood samples were centrifuged promptly (within 5 min), and the resulting supernatant was stored at −80 °C until needed for analysis. The levels of d-ROMs and BAP were determined using a free-radical elective evaluator system (FREE Carrio Duo, Wismerll Co. Ltd., Tokyo, Japan), following the manufacturer’s protocols.

The d-ROM test was employed to measure the organic hydroperoxide concentration, which is indicative of free radical activity, to assess oxidative stress in the plasma. This analysis involved dissolving a 20-μL plasma sample in an acidic buffer (pH 4.8), which released primarily iron ions from the proteins. These ions reacted with the hydroperoxides in the plasma, catalyzing their transformation into alkoxy and peroxy radicals, which, in turn, oxidized an aromatic amine (N, N-diethyl-para-phenylenediamine, 20 μL), forming colored cation radicals. The concentration of these radicals, which is indicative of plasma oxidative capacity, was measured via spectrophotometry at 505 nm. Results were reported in Carratelli Units (U. CARR), where 1 unit is equivalent to 0.08 mg/dL of hydrogen peroxide.

The BAP test quantified the total antioxidant capacity of blood plasma based on its ferric ion reduction ability. For this test, 10 μL of plasma was added to a 50-μL colored solution containing ferric chloride and a thiocyanate derivative. The interaction between the plasma and chromogen-based solution led to ferric ion reduction, causing the solution to decolorize. The intensity of this color change, indicative of the antioxidative properties of the plasma, was quantitatively assessed at 505 nm using a photometer. The degree of discoloration, proportional to the plasma’s ferric ion reduction capability, was used to calculate the BAP test results, which were expressed in micromoles per liter (µmol/L) of reduced ferric ions [29].

Statistical analysis

The sample size was calculated based on a previous study [4] that used comparable methods. We used a standard deviation of 14% and a mean difference (d) of 12% in the rate of change in quadriceps strength before and after surgery (our primary outcome). We determined that 23 participants per group would be sufficient to refute the null hypothesis, with 80% statistical power and a significance level of less than 0.05. Despite our efforts, we were unable to meet the participant recruitment targets.

All statistical analyses were performed using IBM SPSS Statistics for Windows, version 26 (IBM Corp., Armonk, NY, USA). Normal distribution and homogeneity of variance were confirmed using Shapiro-Wilk and Levene’s tests, respectively. Assessments of potential group differences in baseline and surgical data (e.g., age, sex, and tourniquet time) were performed using the chi-square test or independent samples t-test. Group comparisons of changes in quadriceps strength before and after the intervention and surgery, as well as Δpain and swelling in the knee and thigh, were assessed using the independent samples t-test or Mann-Whitney U test, depending on the normality of data distribution of each variable. Two-way repeated-measures analysis of variance (time × group) was performed to compare the changes in measures over time between the groups. The discovery of main or interaction effects led to subsequent post-hoc tests with Bonferroni correction to identify the origin of significant differences. Specifically, in the context of multiple comparisons over time, tests were conducted between baseline and each subsequent time point. During the Pre-Op phase, data collection for d-ROMs and BAP was inadvertently omitted for one individual in the LST group due to oversight by a team member. As a result, the d-ROMs and BAP data for the LST group at the Pre-Op time point were analyzed based on 10 participants. Statistical significance (two-tailed) was set at a p-value of <0.05.

## Results

Table 1 summarizes the demographic and clinical characteristics of participants in both groups. No significant differences in the baseline and surgical data were found between the groups.

**Table 1:** Demographic and clinical characteristics of participants in both groups Variables following a normal distribution are presented as mean values with standard deviations (SDs), whereas those not adhering to a normal distribution are displayed as median values with interquartile ranges (IQRs). No significant differences were observed between any of the items. LST, low-intensity resistance training with slow movements and tonic force generation; BFR, low-load resistance training with blood flow restriction; BMI, body mass index; OA, osteoarthritis; TKA, total knee arthroplasty

Demographic and Clinical Characteristics	LST group (n=11)	BFR group (n=11)
Age (years), median (IQR)	73 (70, 75)	73 (72, 75)
Male, n (%)	2 (18)	2 (18)
Height (m), mean (SD)	1.5 ± 0.1	1.5 ± 0.03
Weight (kg), mean (SD)	58 ± 8	57 ± 7
BMI (kg/m^2^), mean (SD)	25 ± 3	24 ± 2
Number of preoperative interventions, mean (SD)	6 ± 3	7 ± 2
Number of participants who received preoperative intervention at least twice a week, n (%)	4 (36)	4 (36)
Number of postoperative interventions, median (IQR)	9 (9, 10)	11 (9, 13)
Current medical history, n (%)		
Diabetes	0 (0)	1 (9)
Hyperlipidemia	3 (27)	4 (36)
Hypertension	5 (45)	4 (36)
Contralateral knee		
OA and TKA, n (%)	9 (82)	9 (82)
Quadriceps strength (Nm/kg), mean (SD)	1.13 ± 0.25	1.29 ± 0.38
Tourniquet time (min), median (IQR)	56 (53, 59)	58 (52, 64)

In each group, four participants (36%) engaged in preoperative interventions with a minimum frequency of twice a week. The safety data for preoperative LLRT-BFR, presented in Table 2, indicated no significant interactions, and the main effects of time and group were observed for D-dimer and hs-CRP.

**Table 2:** Blood evaluations before and after the intervention and surgery in both groups The levels of D-dimer, high-sensitivity C-reactive protein (hs-CRP), diacron-reactive oxygen metabolites (d-ROMs), and biological antioxidant potential (BAP) were assessed pre- and post-intervention, and the levels of interleukin-6 (IL-6) were evaluated pre- and post-surgery for both groups. Data are provided as mean ± standard deviation. *p<0.05, significantly different from “Pre” within the respective groups. LST, low-intensity resistance training with slow movements and tonic force generation; BFR, low-load resistance training with blood flow restriction.

	LST group (n=11)		BFR group (n=11)		
Parameters	Pre	Post	Pre	Post	p-value (interaction/group/time)
D-dimer (μg/mL)	0.7 ± 0.3	0.7 ± 0.4	1.0 ± 0.6	0.9 ± 0.5	(0.30/0.20/0.56)
hs-CRP (mg/dL)	0.1 ± 0.1	0.3 ± 0.9	0.1 ± 0.1	0.4 ± 1.1	(0.81/0.94/0.23)
d-ROMs (U. CARR)	397 ± 53	379 ± 92	392 ± 42	371 ± 46	(0.73/0.93/0.31)
BAP (µmol/L)	2401 ± 479	2147 ± 207	2349 ± 568	2095 ± 195	(0.97/0.71/0.06)
IL-6 (pg/mL)	1.5 ± 1.0	124 ± 86*	2.0 ± 2.5	191 ± 117*	(0.15/0.15/0.00)

**Figure 2:**
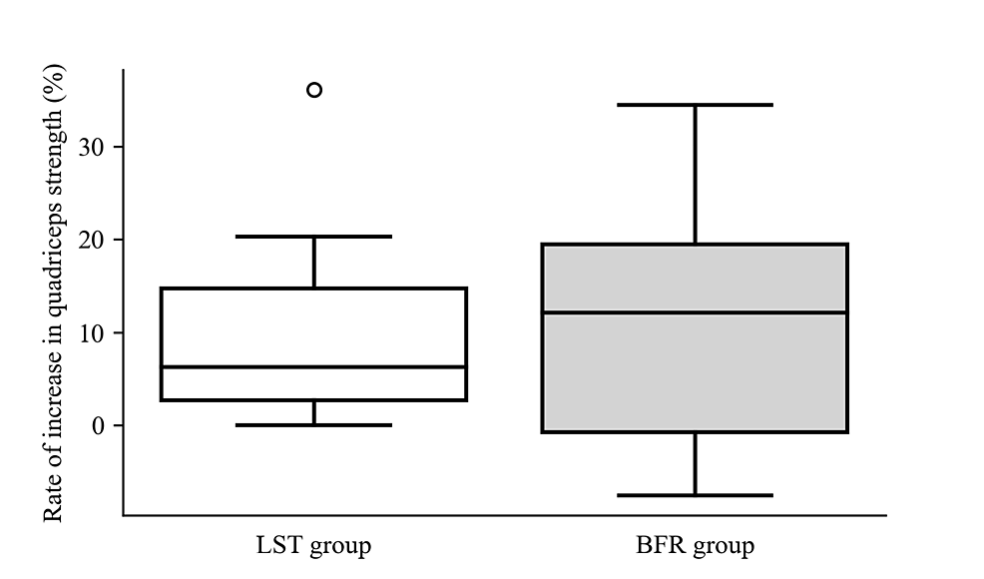
Rate of increase in quadriceps strength before and after the intervention in both groups This boxplot illustrates the median increase in quadriceps strength before and after the two distinct intervention approaches: low-intensity resistance training with slow movements and tonic force generation (LST) and low-load resistance training with blood flow restriction (BFR). The central line in each box represents the median value, while the box’s extents indicate the interquartile range, reflecting the middle 50% of the data. LST is depicted in white and BFR in grey. Whiskers extend from the box to show the range of the data, and any points outside of this range are plotted as individual points, indicating outliers.

**Figure 3:**
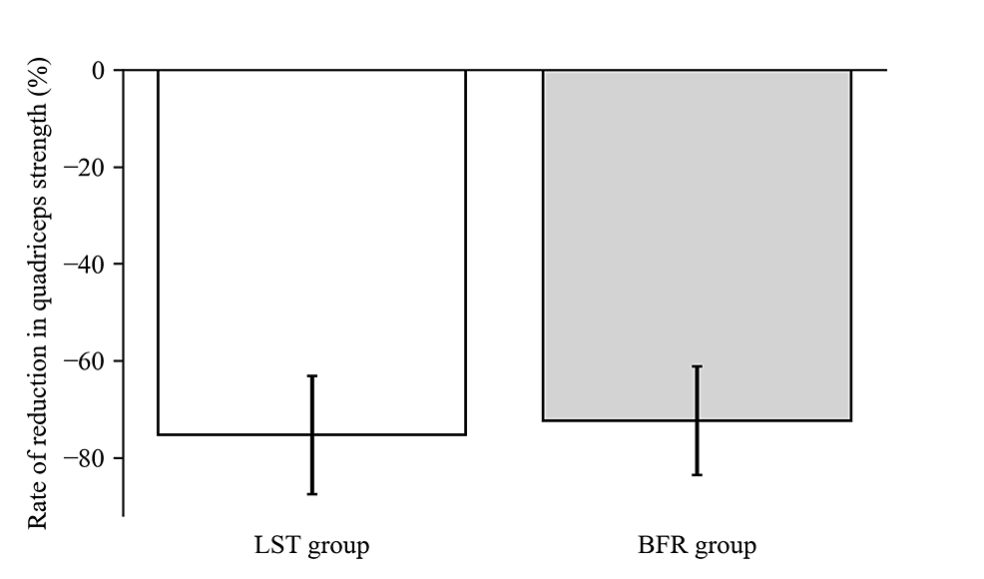
Rate of reduction in quadriceps strength before and after surgery in both groups This bar graph illustrates the average rate of quadriceps strength reduction before and after surgery for two groups of patients scheduled for total knee arthroplasty undergoing two different prehabilitation methods: low-intensity resistance training with slow movements and tonic force generation (LST) and low-load resistance training with blood flow restriction (BFR). The height of each bar indicates the average rate of reduction in quadriceps strength for each group, with LST in white and BFR in grey. The error bars show the standard deviation, highlighting the variability of the reduction rates within each group.

Regarding the primary outcomes, as shown in Figure 2 and Figure 3, no significant differences were observed in the rate of change in quadriceps strength between the two groups before and after the intervention (p>0.99) and before and after surgery (p=0.57). In the LST group, strength increased by 6.2%, with an interquartile range from 2.7% to 14.7% post-intervention and decreased by 75.3% (standard deviation: 12.2%) post-surgery. In the BFR group, strength increased by 12.1%, with an interquartile range from -0.8% to 19.5% post-intervention and decreased by 72.4% (standard deviation: 11.2%) post-surgery.

Table 2 outlines the secondary outcomes, where no significant interaction or main effects related to time or group were detected for d-ROMs or BAP. For IL-6, although no significant interaction and main effects of the group were found, a significant main effect of time was observed. Subsequent post-hoc analysis revealed an increase in IL-6 levels after surgery in both groups. Notably, one individual in the BFR group exhibited elevated pre- and postoperative IL-6 levels (Pre-Op: 9.3 pg/mL, 24 h-Post-Op: 381 pg/mL) and preoperative hs-CRP levels (Pre-Op: 3.6 mg/dL), which were attributed to a cold the patient acquired the day before the surgery. No significant differences were observed in Δpain (LST group: 4.1 ± 3.1, BFR group: 3.7 ± 2.7) or swelling in the knee (LST group: 11.9 ± 3.0%, BFR group: 10.5 ± 2.6%) and thigh (LST group: 7.3 ± 2.5%, BFR group: 6.0 ± 2.4%) between the two groups. Table 3 presents the temporal comparisons between the two groups, including quadriceps strength and knee pain during quadriceps testing, as well as the results of the three performance tests and patient-reported outcomes.

**Table 3:** Temporal changes in physical function, activities of daily living, and quality of life scores in both groups Data are presented as mean ± standard deviation. *p<0.05 shows a significant difference from the baseline within the respective groups. LST, low-intensity resistance training with slow movements and tonic force generation; BFR, low-load resistance training with blood flow restriction; 30-s-CST, the 30-s chair stand test; TUG test, the timed up-and-go test; SCT, the 12-step stair climbing test; KOOS-ADL, daily living activity scores using the Knee Injury and Osteoarthritis Outcome Score; JKOM scores, the Japanese Knee Osteoarthritis Measure scores.

Variables and Time Points	LST group (n=11)	BFR group (n=11)	p-value (interaction/group/time)
				Quadriceps strength (Nm/kg)			(0.66/0.16/0.00)
Baseline	0.94 ± 0.21	1.08 ± 0.39	
1 week before surgery	1.03 ± 0.24	1.20 ± 0.44	
4 days after surgery	0.25 ± 0.14*	0.33 ± 0.15*	
1 month after surgery	0.58 ± 0.15*	0.76 ± 0.29*	
3 months after surgery	0.84 ± 0.16	1.02 ± 0.37	
Knee pain during quadriceps testing			(0.83/0.04/0.00)
Baseline	1.9 ± 1.8	3.2 ± 2.8	
1 week before surgery	1.5 ± 1.9	3.4 ± 2.9	
4 days after surgery	5.6 ± 2.8*	7.1 ± 1.7*	
1 month after surgery	2.1 ± 2.1	4.1 ± 1.0	
3 months after surgery	1.5 ± 1.6	2.7 ± 2.0	
30-s-CST (repetitions)			(0.18/0.73/0.00)
Baseline	12.4 ± 5.3	12.5 ± 4.5	
1 week before surgery	13.8 ± 4.9	15.1 ± 5.0*	
4 days after surgery	8.7 ± 4.4*	7.9 ± 4.0*	
1 month after surgery	13.2 ± 3.4	13.0 ± 4.4	
3 months after surgery	14.5 ± 3.5	15.6 ± 4.4*	
TUG test (seconds)			(0.66/0.55/0.00)
Baseline	8.8 ± 2.2	9.8 ± 3.3	
1 week before surgery	8.5 ± 2.1	8.7 ± 2.0	
4 days after surgery	20.5 ± 8.9*	22.6 ± 10.7*	
1 month after surgery	9.4 ± 1.8	10.1 ± 2.9	
3 months after surgery	8.2 ± 0.9	8.0 ± 1.2	
SCT (seconds)			(0.63/0.71/0.03)
Baseline	25.0 ± 21.5	29.7 ± 24.6	
1 week before surgery	21.8 ± 13.8	22.8 ± 12.6	
1 month after surgery	26.7 ± 8.1	28.7 ± 12.2	
3 months after surgery	19.4 ± 6.3	20.2 ± 7.3*	
KOOS-ADL (%)			(0.97/0.21/0.00)
Baseline	68.2 ± 15.1	61.9 ± 17.2	
1 week before surgery	74.2 ± 10.9	68.3 ± 14.1	
1 month after surgery	70.5 ± 17.3	66.8 ± 9.5	
3 months after surgery	82.4 ± 8.7*	76.3 ± 8.8*	
JKOM scores (points)			(0.98/0.17/0.00)
Baseline	35.6 ± 12.0	43.1 ± 19.1	
1 week before surgery	30.6 ± 11.4	38.5 ± 17.5	
1 month after surgery	33.6 ± 15.4	40.4 ± 17.0	
3 months after surgery	18.2 ± 7.5*	24.5 ± 9.9*	

The analysis revealed no significant interactions between measurements. While group effects, except for knee pain during quadriceps testing, were mostly insignificant, significant time effects were observed for all variables. Furthermore, post-hoc analyses of knee pain during quadriceps testing showed no significant differences between the groups at any of the assessed time points (p>0.05). Additionally, post-hoc tests of the temporal changes in measures for both groups revealed that all measures were equivalent to or improved from baseline at 3 M-Post-Op. The 30-s-CST and SCT in the BFR group and KOOS-ADL and JKOM scores in both groups showed statistically significant improvements from baseline (all p<0.05).

## Discussion

In this study, we assessed the safety and effects of implementing a four-week preoperative LLRT-BFR program on quadriceps strength before and after TKA. In the comparison between BFR and LST groups, no significant differences were observed in the rate of increase in quadriceps strength before and after the intervention or in the rate of reduction in quadriceps strength before and after surgery. Additionally, no significant differences were observed in the temporal changes in quadriceps strength between the groups before and after surgery. These findings suggest that LLRT-BFR may not offer a considerable advantage over LST in improving quadriceps strength during the pre- and postoperative phases. We found no significant changes in the D-dimer and hs-CRP levels before and after the intervention in either the BFR or LST groups, indicating that the risk of thrombosis or systemic inflammation had not increased. This result is noteworthy considering concerns regarding the safety of LLRT-BFR in patients undergoing TKA.

Our analysis revealed no notable differences in the rate of increase in quadriceps strength and temporal changes in antioxidant capacity and oxidative stress levels between the two groups before and after the intervention. The decreased frequency of interventions may have been a significant factor in these findings. Moreover, the BFR and LST groups participated in exercise programs designed to elicit hypoxic stimuli in the targeted muscles. Exercise protocols, including exercise types and sets, were standardized across both groups to ensure comparable exposure to exercise stimuli. Given that the exercise stimuli were identical in both groups, varying the level of hypoxic exposure was necessary to identify any clear differences between the groups. Although LLRT-BFR and LST employ different hypoxia-inducing methods, with LLRT-BFR using external pressure cuffs [17,18] and LST relying on sustained muscle contractions [14,15], the expected disparity in hypoxic stimuli per session is likely minimal. Consequently, enhancing the duration and frequency of interventions is crucial. Although preliminary studies suggested extending interventions to 12 weeks, practical constraints compelled us to shorten the duration to four weeks for this study. To compensate for this, we implemented a relatively higher frequency and volume of sessions.

Unfortunately, the anticipated training frequency was substantially compromised, largely because of the coronavirus disease 2019 (COVID-19) pandemic, which resulted in more participants engaging in less-frequent sessions than planned. This reduced frequency likely limited the capacity to induce a discernible difference in hypoxic exposure between the groups. This may explain the lack of significant variance in the rate of increase in quadriceps strength and temporal changes in antioxidant capacity and oxidative stress levels between the two groups before and after the intervention. Furthermore, the actual sample size in our study was smaller than that initially planned, which likely influenced the outcomes. The anticipated number of participants was significantly affected, primarily due to the COVID-19 pandemic, leading to a reduction in the overall sample size. Additionally, the stringent exclusion criteria for selecting participants contributed to the smaller sample size. Given the specific exercise modality involving blood flow restriction during exercise, we applied strict exclusion criteria. The consequent reduction in the number of participants might have further constrained our ability to detect significant differences between the groups, adding to the challenges posed by the decreased intervention frequency.

The absence of a significant difference in the rate of reduction in quadriceps strength before and after surgery between the BFR and LST groups may be attributed to the inability of the patients in the BFR group to enhance their antioxidant capabilities before surgery compared to those of patients in the LST group. Quadriceps weakness immediately after TKA is associated with knee pain and swelling in the knee and thigh caused by surgical trauma and tourniquet-induced IR injury [1,9,10]. Enhancements in antioxidant capacity through exercise and ischemic preconditioning mitigate acute inflammation caused by IR injuries [13,20]. In this study, no significant improvements in antioxidant capacity were observed in the BFR group compared with those in the LST group before surgery. This could account for the absence of significant differences in the temporal changes in IL-6 levels before and after surgery, Δknee pain, and swelling in the knee and thigh between the groups. Furthermore, these findings may explain the lack of effective mitigation of quadriceps weakness before and after surgery in the BFR group compared to that in the LST group.

As mentioned previously, no significant differences were observed in the rate of increase in quadriceps strength before and after the intervention or in the rate of reduction in quadriceps strength before and after surgery between the groups. This suggests that, by the fourth postoperative day, quadriceps strength in the BFR group was not significantly higher than that in the LST group. Given that no significant difference was observed in quadriceps strength at baseline between the groups and that both groups underwent the same postoperative intervention, the absence of significant differences in the temporal changes in quadriceps strength pre- and post-surgery between the groups was anticipated. This aspect is crucial, as previous research has established a correlation between quadriceps strength, functional outcomes, and patient satisfaction after TKA [3,5,30]. Consequently, the absence of significant differences in the temporal changes in quadriceps strength before and after surgery between the groups likely contributed to the absence of notable differences in functional performance tests (30-s-CST, TUG test, and SCT), as well as in patient-reported outcome measures, including the KOOS-ADL and JKOM.

Study limitations

This study had some limitations, including the impact of the COVID-19 pandemic on intervention frequency. Additionally, lower-than-expected participant numbers due to pandemic-related recruitment difficulties and the implementation of stringent exclusion criteria may limit statistical robustness, particularly regarding safety markers, such as D-dimer and hs-CRP levels. Although D-dimer and hs-CRP levels were monitored, a broader selection of safety biomarkers may offer a more comprehensive safety assessment. The single-blind design implied that investigators were unaware of group assignments; however, participants might have inferred the group assignments due to noticeable pressure differences in the cuffs (20 mmHg vs. 100-120 mmHg), potentially affecting engagement. Our use of stringent exclusion criteria for selecting participants with specific preoperative fitness and health conditions limits the broader applicability and generalization of the results.

## Conclusions

Our study demonstrated that a four-week preoperative LLRT-BFR program does not significantly outperform LST in enhancing quadriceps strength before and after TKA. However, both interventions appear to be safe. Future research should aim to overcome the limitations encountered in this trial, possibly by extending the intervention duration, increasing the sample size, and ensuring higher intervention adherence, to more comprehensively elucidate the potential benefits of LLRT-BFR in the preoperative conditioning of patients undergoing TKA.
